# The aggrephagy-related gene TUBA1B influences clinical outcomes in glioma patients by regulating the cell cycle

**DOI:** 10.3389/fonc.2025.1531465

**Published:** 2025-02-28

**Authors:** Zesheng Sun, Pengcheng Huang, Jialiang Lin, Guiping Jiang, Jian Chen, Qianqian Liu

**Affiliations:** ^1^ Department of Neurosurgery, Affiliated Hospital of Nantong University, Medical School of Nantong University, Nantong, Jiangsu, China; ^2^ Tianjin Medical University, General Hospital, Tianjin, China

**Keywords:** glioma, aggrephagy, TUBA1B, prognostic marker, immune microenvironment

## Abstract

**Background:**

Gliomas are common primary malignant brain tumors, with glioblastoma (GBM) being the most aggressive subtype. GBM is characterized by high recurrence rates and treatment resistance, leading to poor patient outcomes. Current prognostic models have limited predictive power, underscoring the need to elucidate underlying mechanisms and identify novel biomarkers to improve therapeutic strategies and prognostic models.

**Methods:**

Gene expression and clinical data for GBM and LGG were obtained from the TCGA and CGGA database, while single-cell sequencing data from GSE167960 were selected from the GEO database. Molecular characteristics of gliomas were revealed through normalization, consensus clustering analysis, immune scoring, cell infiltration analysis, and pathway analysis. TUBA1B, identified as a key gene through machine learning, was incorporated into a nomogram model using multivariate Cox regression. Its functions were validated through qRT-PCR, *in vitro* functional assays, and mouse xenograft models. All data analyses and statistics were performed using R software.

**Results:**

Consensus clustering of the TCGA glioma dataset identified two aggrephagy subtypes (C1 and C2), with C2 showing worse survival outcomes and higher immune infiltration. TUBA1B was identified as an independent prognostic marker, with high expression associated with upregulated cell cycle pathways and alterations in the immune microenvironment. TUBA1B was shown to influence glioma cell proliferation, migration, invasion, and autophagy, impacting tumor progression and treatment response through intercellular communication and metabolic pathways.

**Conclusion:**

The study demonstrates that high TUBA1B expression is closely associated with glioma malignancy and poor prognosis, making it a potential therapeutic target.

## 1 Introduction

An estimated 40% of all brain tumors are gliomas, which are the most common primary malignant brain tumors (1). Among them, glioblastoma (GBM) is the most aggressive subtype. According to the World Health Organization (WHO) classification, gliomas are categorized into four histopathological grades: I, II, III, and IV. GBM (WHO grade IV) is the most invasive subtype, characterized by neovascularization, and WHO grades II and III are considered lower-grade gliomas (LGG) (2, 3). Histologically, gliomas exhibit high cellular density, active mitosis, vascular proliferation, and necrosis (4). Due to the aggressive nature of the tumor and resistance to chemotherapy and radiotherapy, patients often face high recurrence rates and functional impairments (5). Gliomas are currently treated with surgical resection, adjuvant chemotherapy, and radiotherapy. The median survival for LGG patients can range from five to ten years with combination therapies, while GBM patients typically have a median survival of only one to two years (6, 7). The prognosis of glioma patients varies significantly and is influenced by factors such as tumor grade, isocitrate dehydrogenase (IDH) mutation (8), and epidermal growth factor receptor (EGFR) amplification (9). Current glioma prognosis models are mainly based on clinical factors, but their predictive capacity is limited (10–13). Therefore, it is urgently needed to discover the mechanisms underlying glioma genesis and to identify biomarkers for improving therapeutic strategies and prognostic models.

Aggrephagy is a selective form of autophagy responsible for degrading misfolded or aggregated proteins (e.g., those generated by genetic mutations or cellular stress), which are typically recognized as damaged or misfolded within cells and need to be eliminated to prevent their accumulation (14). Aggrephagy plays a crucial role in cellular homeostasis by removing protein aggregates that might otherwise accumulate and disrupt cellular function. These misfolded proteins may arise continuously within cells due to genetic mutations, incomplete mRNA translation, post-translational misfolding, improper protein modifications, and oxidative stress (15). While these misfolded proteins are typically degraded via the ubiquitin-proteasome system (UPS), in certain cases (such as during protein aggregation), UPS may fail to degrade the target proteins effectively (16). Under these circumstances, aggrephagy becomes an alternative pathway for protein degradation. Aggrephagy is important in maintaining cellular homeostasis and is implicated in various human diseases, including neurodegenerative disorders, cataracts, and type II diabetes (17, 18).

Autophagy plays a dual role in tumorigenesis: at low levels, autophagy can facilitate the initial stages of cancer progression by providing energy and promoting cellular adaptation to stress. However, at elevated levels, autophagy enables tumor cells to survive under nutrient-scarce conditions by maintaining cellular homeostasis and removing damaged components, such as aggregated proteins, thus contributing to tumor cell survival in the later stages of cancer progression (18). Despite high autophagy-related gene expression and activity in glioma tissues and cells (16, 19, 20), whether aggrephagy exerts a similar regulatory role in glioma remains largely uninvestigated. As a result, the study of aggrephagy in gliomas is of considerable academic and clinical interest.

In this study, we explore the role of aggrephagy and its key gene, TUBA1B, in glioma, uncovering its multiple impacts on the tumor microenvironment. Through clustering analysis of The Cancer Genome Atlas (TCGA) dataset, we found that a high level of TUBA1B expression in gliomas indicates a poor prognosis and a rapid progression of the disease. Elevated TUBA1B expression promotes cell proliferation and migration and significantly affects the cell cycle, autophagy, and apoptosis. Immunological analysis indicates that TUBA1B is linked to cancer-associated fibroblasts and various immune cell infiltrations, implying its involvement in modulating the tumor microenvironment and intercellular communication. Furthermore, high TUBA1B expression is correlated with enhanced tumor stemness and decreased sensitivity to immunotherapy in glioma. These findings not only enhance our understanding of aggrephagy in glioma but also provide potential directions for developing new therapeutic targets with important clinical implications.

## 2 Methods

### 2.1 Processing and collection of data

In this study, we obtained data on glioblastoma and lower-grade gliomas (GBM and LGG) from the TCGA database. The gene expression data underwent log_2_(TPM+1) transformation to standardize the data and mitigate the effects of sequencing depth and gene length. Additionally, corresponding clinical data were acquired. During data curation, samples lacking survival data were excluded, resulting in a final cohort of 660 samples with complete expression profiles and clinical information.

Additionally, we incorporated glioma data from the Chinese Glioma Genome Atlas (CGGA) database to further validate our findings. The CGGA database includes three mRNAseq data (mRNAseq_301, mRNAseq_325 and mRNAseq_693). Gene expression data from these cohorts were processed similarly to the TCGA and GEO datasets, with log_2_(TPM+1) transformation to standardize the data. The clinical data for these samples were also curated, and only samples with complete survival and clinical information were included.

For single-cell sequencing results, we selected the single-cell sequencing dataset GSE167960 from the Gene Expression Omnibus (GEO) database, which includes six samples. We performed data normalization and quality control to remove outliers or samples with low cell counts, as well as annotated cell types based on gene expression characteristics.

Subsequently, we integrated the multi-sample data from TCGA with the single-cell data from GEO. Through survival analysis, differential gene expression analysis, and cellular heterogeneity analysis, we systematically explored the molecular characteristics of gliomas to identify potential prognostic biomarkers and key molecular pathways. These analyses provide an essential foundation for elucidating the relationships between different molecular subtypes and their roles within the tumor microenvironment.

### 2.2 Consensus clustering analysis

To assess the differential expression of autophagy-related genes across glioma patients, we applied consensus clustering (CC) to classify the patients (21). Initially, we divided all samples based on a range of cluster numbers (k = 2-9). We then calculated the consensus score matrix and plotted CDF curves along with Delta area plots to determine the optimal number of clusters. The optimal k value was then selected for further analysis.

### 2.3 Immune scoring and immune checkpoint analysis

For immune infiltration analysis, we used multiple algorithms, including TIMER, CIBERSORT, MCP-counter, and xCell. These bioinformatics tools apply different algorithms to infer and quantify the relative proportions of various immune cell types in tumor samples based on gene expression data. TIMER is a tool for estimating the abundance of immune cells from RNA-seq data, while CIBERSORT uses a deconvolution algorithm to estimate the fraction of immune cells in a mixed tissue sample.

### 2.4 Cell infiltration analysis

To comprehensively analyze the cell types within the tumor microenvironment, we employed multiple bioinformatics tools, including TIMER, CIBERSORT, MCP-counter, and xCell. Each tool applies distinct algorithmic principles to infer and quantify the relative proportions of various immune cell types within tumor samples. The analysis involved importing gene expression data, running the “IOBR” package, and organizing the output to reveal the infiltration characteristics of different cell types. These insights provide a detailed understanding of the cellular composition of the tumor microenvironment.

### 2.5 Pathway analysis

In the pathway analysis, we utilized Gene Ontology (GO) and Kyoto Encyclopedia of Genes and Genomes (KEGG) to explore the functions and pathways associated with differentially expressed genes. Additionally, Gene Set Enrichment Analysis (GSEA) was employed to investigate the enrichment of differentially expressed genes within known pathways, aiding in the identification of functional and significant pathways. During the analysis, we input a filtered set of genes with differential expression (criteria: adjusted P < 0.05, log_2_FC > 1) and compared them against reference pathway sets from the Reactome or KEGG databases to identify highly correlated pathways. The activity levels of various biological pathways were assessed by calculating enrichment scores and conducting statistical tests, which helped elucidate these pathways’ potential roles in tumor biology.

### 2.6 Selection of TUBA1B

To identify the key gene TUBA1B, the study employed three machine learning methods: LASSO regression, random forest, and support vector machine (SVM). LASSO regression is a method that applies a penalty to reduce the number of candidate genes, effectively narrowing down the gene list. Random forest, a decision tree-based algorithm, evaluates gene importance scores by constructing multiple decision trees and selecting the most influential genes based on their contribution to the classification. SVM is used to classify candidate genes and validate their classification performance. By cross-analyzing the results from these methods, TUBA1B was identified as a key gene associated with autophagy in gliomas.

### 2.7 The construction and evaluation of the nomogram

Our nomogram was constructed using univariate and multivariate Cox regression analyses to identify independent prognostic factors significantly associated with survival. A nomogram provides a visual representation of patient survival probabilities, helping clinicians predict survival rates at 1 year, 3 years, and 5 years based on multiple factors. To assess the predictive performance of the nomogram, several statistical methods were used. Receiver Operating Characteristic (ROC) curves were plotted to evaluate the model’s ability to discriminate between patients with different survival outcomes. The area under the curve (AUC) was calculated at 1-year, 3-year, and 5-year intervals to measure the accuracy of the model. Calibration curves were then constructed to compare the predicted survival probabilities with the actual observed outcomes, evaluating the degree of agreement between them. A closer alignment between predicted and observed survival indicates a better model performance. Finally, Decision Curve Analysis (DCA) was performed to assess the clinical net benefit of the nomogram. DCA evaluates whether the model provides a net benefit compared to a strategy of treating all patients or treating none. This method helps to identify the most clinically relevant thresholds for the model’s decision-making. These comprehensive evaluations demonstrated the nomogram’s superiority in survival prediction, supporting its clinical application.

### 2.8 RNA extraction and quantitative RT-PCR

TRIzol reagent (Invitrogen, Carlsbad, CA) was used to isolate total RNA from transfected and control cell samples. The qRT-PCR reactions were performed in triplicate using Taq Pro Universal SYBR qPCR Master Mix (Vazyme Biotech Co., Ltd., Nanjing, Jiangsu, China) as directed by the manufacturer. The experiment utilized TUBA1B-specific primers, including the forward primer 5′-GAGCAGCTCATCACAGGCATT-3′ and reverse primer 5′-TGCCTGTGATGAGCTGCTCTT-3′. A 2^−ΔΔCt^ method, normalized to GAPDH as an internal control, was used to determine the relative expression of TUBA1B following qRT-PCR.

### 2.9 Culturing and transfecting cells

Human glioma U251 and U87 cell lines were cultured and maintained in DMEM (Gibco, Grand Island, NY, USA) supplemented with 10% fetal bovine serum (FBS, Gibco, USA) and 1% penicillin-streptomycin solution in an incubator at 37°C with 5% CO_2_. To explore the functional complexity of TUBA1B in GBM, TUBA1B was knocked down in U251 and U87 cells using negative control shRNA (shNC) and shTUBA1B. The transfection was performed using Lipofectamine 3000 reagent (Invitrogen, Carlsbad, CA, USA) strictly according to the manufacturer’s protocol. Transfection was initiated when U251 and U87 cells reached approximately 70%-80% density in 6-well plates. Transfected cells were incubated under standard conditions for 48-72 hours to ensure effective knockdown of TUBA1B.

### 2.10 *In vitro* functional experiments

#### 2.10.1 Cell proliferation

Following TUB1B knockdown, glioma cell proliferation was assessed using the CCK-8 assay. Following transfection with TUBA1B-specific shRNA for 48 hours, cells were harvested and counted. In 96-well plates, cells were seeded at a density of 2×10³ per well. At 37°C, 10 μL of CCK-8 reagent (Dojindo, Japan) was added every 24 hours and incubated for 2 hours. The proliferation of cells was evaluated by measuring absorbance at 450 nm using a microplate reader.

#### 2.10.2 Migration and invasion

The Transwell assay assessed cell migration and invasion after TUBA1B knockdown. After 48 hours post-transfection, cells were harvested and counted, then seeded at a density of 5×10^4^ per well in the upper chamber of 24-well Transwell inserts (8 μm pore size, without Matrigel, Corning, USA). The lower chamber was filled with a complete medium supplemented with 20% FBS. The upper chamber was swabbed with cotton swabs after 24 hours, and the cells were stained with crystal violet and fixed with 4% paraformaldehyde. Invasion assays followed similar procedures, utilizing Matrigel-coated inserts, seeding 8×10^4^ cells per well, and incubating for 48 hours to assess the number of invasive cells.

#### 2.10.3 Cell cycle analysis

Flow cytometry analyzed cell cycle distribution following TUBA1B knockdown. We harvested the cells 48 hours post-transfection, washed them with PBS, and incubated them for 15 minutes in RNase-containing PBS containing PI reagent (BD Biosciences, USA). Cells were analyzed using a flow cytometer, and data were processed with ModFit LT software.

### 2.11 Western blot analysis

Using RIPA lysis buffer (containing protease inhibitors, Beyotime, China), total protein was extracted from treated cells. Protein concentrations were determined via the BCA method. To conduct electrophoresis, 30-50 μg of protein were loaded onto 6% or 10% SDS-polyacrylamide gels. Separated proteins were transferred to methanol activated PVDF membranes (Millipore, USA). Membranes were blocked with 5% BSA (Sigma-Aldrich, USA) in TBST at room temperature for 1 hour. An overnight incubation at 4°C with primary antibodies was performed on membranes: anti-LC3B (1:1000, Cell Signaling Technology, USA), anti-p62 (1:1000, Proteintech, USA), anti-Bcl-2 (1:1000, Cell Signaling Technology, USA), anti-Cyclin D1 (1:1000, Cell Signaling Technology, USA), and loading control anti-Tubulin 1 (1:5000, Proteintech, USA). Following three 10-minute TBST washes, membranes were incubated for 1 hour at room temperature with HRP-conjugated secondary antibodies (anti-rabbit or anti-mouse IgG, 1:5000, Cell Signaling Technology, USA), followed by three additional 10-minute TBST washes. Protein bands were developed with ECL-plus™ chemiluminescent kit (Thermo Fisher, USA) and visualized using a chemiluminescence imaging system.

### 2.12 *In vivo* xenograft mouse experiments

We obtained female BALB/c nude mice from the Animal Laboratory at Nantong University Medical College, aged 4 weeks. Well-growing U251 cells were prepared and transiently transfected with control and TUBA1B knockdown siRNA. Trypsinization and PBS washing were performed after 24 hours. The cells were counted and diluted to a concentration of 5 × 10^6^ cells/100 μL. Under respiratory anesthesia, 100 μL of control/TUBA1B knockdown cells were subcutaneously injected into the mice. The growth of subcutaneous tumors was monitored. After 28 days, *in vivo* imaging experiments were conducted to measure tumor size. In accordance with animal welfare guidelines, this animal study was approved by the Animal Ethics Committee of Nantong University Medical College (S20240116-009).

### 2.13 Tumor stemness and immunotherapy benefit analysis

Six tumor stemness indices were utilized: DMPss (differentially methylated probes), DNAss (DNA methylation), ENHss (enhancer elements/DNA methylation), EREG.EXPss (epigenetically regulated RNA expression), EREG-METHss (epigenetically regulated DNA methylation), and RNAss (RNA expression). Spearman analysis was performed to explore the correlation between stemness characteristics and TUBA1B expression (22). Tumor Immune Dysfunction and Exclusion (TIDE) was applied to predict the response to immune checkpoint blockade therapy (23).

### 2.14 Analyses of single-cell sequencing

Single cell sequencing data from multiple samples were collected and processed using the “Seurat” package for quality control and normalization. UMAP dimensional reduction was applied to perform clustering analysis, identifying different cell groups. Known cell markers were used to classify cells into eight groups: Oligodendrocytes, Macrophages, Glioma cells, Endothelial cells, Monocytes, T cells, Pericytes, and B cells. The “AddModuleScore” package was employed for gene set variation analysis to evaluate the autophagy-related gene expression levels in different cell groups. The expression of TUBA1B was analyzed across these cell groups, focusing on its distribution in Oligodendrocytes, Macrophages, Glioma cells, and Pericytes. Using the “CellChat” package, cell communication analysis was performed to explore communication patterns among cell groups with high TUBA1B expression, with particular attention to interactions with other cell groups. Signal pathway enrichment analysis was conducted using “cellchat” to identify the main input and output signaling pathways. Additionally, the “scMetabolism” package was used for in-depth analysis of metabolism pathways related to Glioma cells, identifying associations with starch and sucrose metabolism, propionate metabolism, oxidative phosphorylation, fatty acid degradation, and butyrate metabolism. These steps helped reveal the potential mechanisms by which TUBA1B regulates glioma cell biological behavior through intercellular communication and metabolic pathways in the tumor microenvironment.

### 2.15 Statistical analysis

The research data was analyzed statistically using R software (version 4.3.1). Data were evaluated using the Shapiro-Wilk test to determine whether they were normally distributed. Students’ t-tests and one-way ANOVAs were conducted to compare two groups and multiple groups of normally distributed variables. The Wilcoxon test was used for comparisons between two groups of non-normally distributed data, and the Kruskal-Walli test was used for comparisons among multiple groups. Survival analysis was performed using the Kaplan-Meier method, which estimates the probability of survival over time. Log-rank tests were used to compare the survival distributions between groups. The Kaplan-Meier method generates survival curves, and the log-rank test assesses whether there are statistically significant differences between these curves. To ensure the robustness of the findings, the Cox proportional hazards regression model was applied for multivariate analysis, adjusting for potential confounders such as age, gender, and clinical features. The hazard ratio (HR) and corresponding 95% confidence interval (CI) were calculated to evaluate the risk of death associated with each variable. A statistically significant difference was considered when P < 0.05.

## 3 Results

### 3.1 Biological characteristics and clinical significance of aggrephagy subtypes in glioma

**Figure 1:**
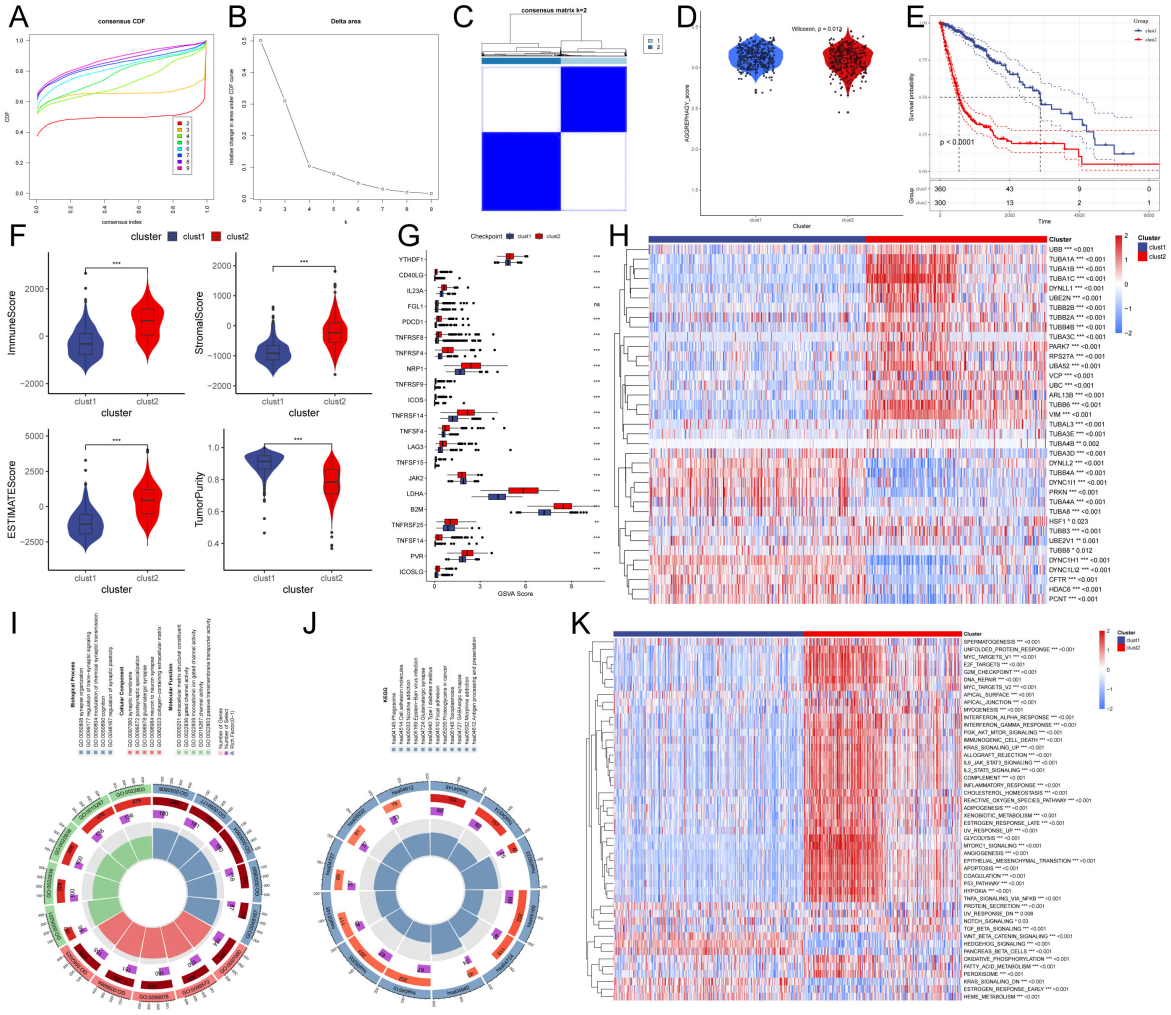
Characteristics of autophagy subtypes in glioma. **(A, B)** Cumulative distribution function (CDF) curves and Delta area plots for consensus scores of autophagy subtypes in the TCGA (GBM+LGG) dataset. **(C)** Consensus score matrix for all samples when k = 2. A higher consensus score between two samples indicates a higher likelihood of their co-clustering in different iterations. **(D)** Autophagy scores of the two clusters. **(E)** Kaplan-Meier survival curves for the two clusters. **(F)** Comparison of Immune score, Estimate score, Stromal score, and Tumor purity between the two clusters. **(G)** Expression of immune checkpoint genes between the two clusters. **(H)** Heatmap showing differentially expressed genes between the two clusters. **(I, J)** Gene Ontology (GO) and Kyoto Encyclopedia of Genes and Genomes (KEGG) pathway analysis of differentially expressed genes between the two groups. **(K)** Heatmap of Gene Set Enrichment Analysis (GSEA) for differentially expressed genes between the two groups.

In order to investigate aggrephagy’s potential role in glioma, we performed aggrephagy subtype classification using the ConsensusClusterPlus algorithm on the TCGA dataset (GBM+LGG). All samples were divided into k (k = 2–9) clusters. By analyzing the cumulative distribution function (CDF) curve, and Delta area plot, we identified k = 2 as the optimal number of subtypes (**Figures 1A**–**C**). Aggrephagy scores were significantly different between the two clusters, with patients in the C2 cluster showing worse survival outcomes (**Figures 1D, E**). To further understand the immunological differences between the two clusters, we employed multiple algorithms to assess immune infiltration, including TIMER, CIBERSORT, MCP-counter, xCell, Immune checkpoints, and ESTIMATE. The results revealed that overall immune infiltration was notably greater in the C2 cluster (**Figures 1F, G**, **Supplementary Figure 1**). Based on this, we defined the C1 cluster as “immune-cold” tumors and the C2 cluster as “immune-hot” tumors. Further analysis of key differentially expressed genes revealed that multiple tubulin-related genes (such as TUBA1A, TUBA1B, TUBA1C, TUBA3C, TUBA4B, TUBA3E, TUBA3D, TUBA4A, and TUBA8) were significantly altered in the C2 cluster compared to the C1 cluster. In addition, genes associated with protein degradation and stress response (such as UBB, UBC, UBA52, RPS27A, VCP, and HSF1) also showed significant changes (**Figure 1H**). Pathway and functional analysis of these differentially expressed genes indicated their involvement in cell adhesion molecules and trans-synaptic signaling regulation. Specifically, the “immune-hot” tumors exhibited significant upregulation in several pathways, including cell cycle, proliferation, metabolism, signaling, immune regulation, and stress response pathways, such as MYC Targets, E2F Targets, G2M Checkpoint, Interferon Alpha/Gamma Response, Inflammatory Response, TNFA Signaling via NFKB, PI3K/AKT/mTOR Signaling, IL6 JAK/STAT3 Signaling, and Glycolysis (**Figures 1I–K**). These results suggest that the C2 cluster not only Contributes significantly to maintaining cellular functions and responding to external stimuli but also that its extensive pathway activity may have significant implications for glioma progression.

### 3.2 TUBA1B as a key aggrephagy gene and independent prognostic marker for glioma patients

**Figure 2:**
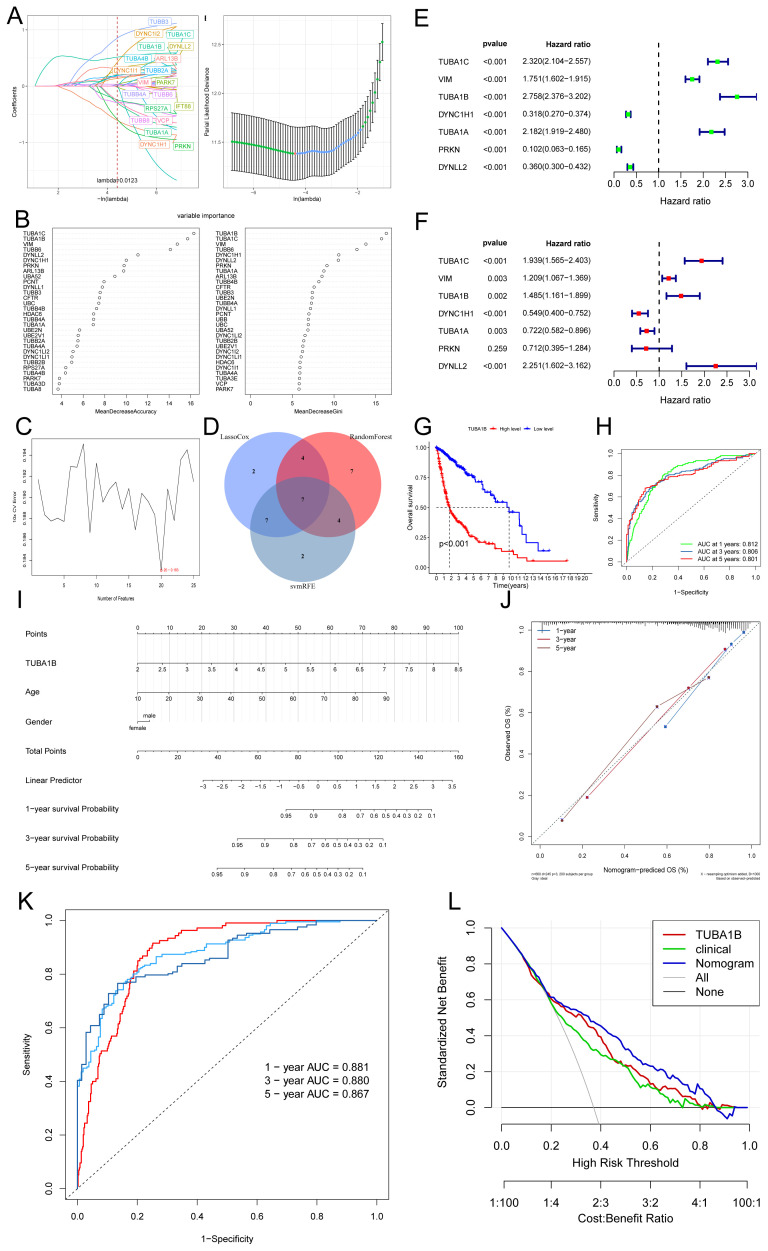
TUBA1B as an independent prognostic marker in glioma patients. **(A)** The relationship between partial likelihood deviance and log(λ) in the LASSO Cox regression model. The lambda parameter represents the coefficient of a feature. The x-axis shows the influence of lambda on the independent variables, while the y-axis represents the coefficient of the independent variables. **(B)** Random Forest results. **(C)** Support vector machine (SVM) curve results. **(D)** Venn diagram of key genes identified through the intersection of three machine learning methods. **(E, F)** Univariate and multivariate Cox regression analysis of seven key genes. **(G)** Kaplan-Meier survival analysis of glioma patients stratified by high and low TUBA1B expression. **(H)** Receiver operating characteristic (ROC) curves predicting 1-, 3-, and 5-year prognosis of glioma patients based on TUBA1B expression. **(I)** Nomogram constructed using TUBA1B expression and various clinical characteristics. **(J)** Calibration curves of the nomogram for 1-, 3-, and 5-year overall survival probabilities. **(K)** ROC curves demonstrating the predictive value of the nomogram for 1-, 3-, and 5-year survival in glioma patients. **(L)** Decision curve analysis (DCA) curves comparing the predictive performance of the nomogram.

To identify key genes associated with aggrephagy in glioma, we employed three machine learning methods: LASSO regression, random forest, and support vector machine, to narrow down candidate genes (**Figures 2A–C**). Through cross-analysis, we identified seven aggrephagy-related common genes: TUBA1C, VIM, TUBA1B, DYNC1H1, TUBA1A, PRKN, and DYNLL2 (**Figure 2D**). A subsequent univariate and multivariate Cox regression analysis found that, except for PRKN, all of the remaining genes played independent prognostic roles. Among them, TUBA1C, VIM, TUBA1B, and TUBA1A were confirmed as risk factors, while DYNC1H1 and DYNLL2 were considered protective factors (**Figures 2E, F**). We identified TUBA1B and TUBA1C as the most significant prognostic genes in glioma by integrating results from random forest, univariate, and multivariate Cox regression analyses, highlighting their high weights. It has been shown that TUBA1C regulates the cell cycle and is associated with poor prognoses in glioma cells (24). Therefore, we chose to investigate the other gene, TUBA1B. In the TCGA database, Kaplan-Meier curve analysis showed that patients with low TUBA1B expression had significantly better prognoses than those with high expression. The low-expression group exhibited a significantly longer survival time compared to the high-expression group, suggesting that elevated TUBA1B expression may correlate with poor prognosis (**Figure 2G**). In addition to the TCGA and GEO datasets, we further validated our findings using data from the CGGA database, which includes a broader data of glioma samples. As shown in **Supplementary Figure 2**, our analysis of CGGA data confirmed the significant association between TUBA1B expression and poor prognosis in glioma patients. These results, consistent with our findings from the TCGA and GEO datasets, reinforce the robustness and relevance of TUBA1B as a potential prognostic biomarker in glioma. According to the ROC curve analysis, TUBA1B has AUC values of 0.812, 0.806, and 0.801 for predicting 1-year, 3-year, and 5-year survival, respectively (**Figure 2H**). To facilitate the clinical application of TUBA1B as a prognostic marker, we constructed a nomogram incorporating various clinicopathological factors, including TUBA1B expression, to better predict overall survival rates for glioma patients (**Figure 2I**). Calibration curves, ROC curves, and DCA were used to evaluate this model. Calibration curves showed that the nomogram’s predictions of survival after a year, three years, and five years were very close to the actual outcomes (**Figure 2J**). Based on the ROC curve analysis, the nomogram’s AUC values for predicting 1-year, 3-year, and 5-year survival were 0.881, 0.880, and 0.867, respectively (**Figure 2K**). A DCA revealed that the nomogram had a higher clinical net benefit between 20% and 80% (**Figure 2L**). Based on these findings, the nomogram is more accurate in predicting glioma patient survival than any single diagnostic feature, highlighting the potential of TUBA1B as a valuable prognostic biomarker.

### 3.3 Pathway analysis of TUBA1B-related genes

**Figure 3:**
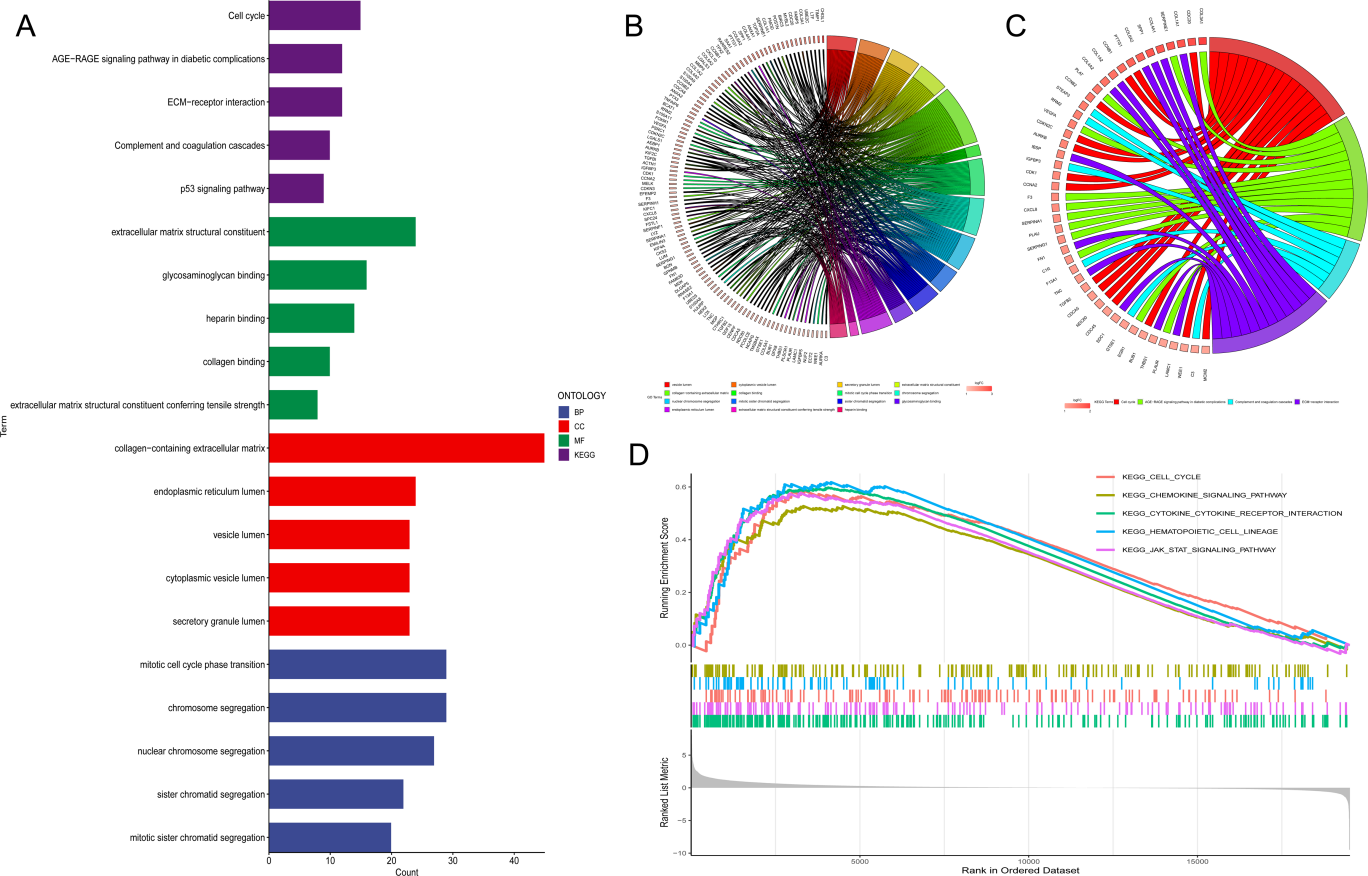
Pathway analysis of genes associated with TUBA1B expression. **(A−C)** Gene Ontology (GO) and KEGG pathway analysis of upregulated differentially expressed genes (DEGs) in the high and low TUBA1B expression groups. **(D)** Gene Set Enrichment Analysis (GSEA) of upregulated DEGs in the high and low TUBA1B expression groups.

We divided the TCGA database into high and low TUBA1B expression groups based on the median expression level in order to examine TUBA1B’s role in gliomas. We identified differentially expressed genes between the two groups and concentrated on those upregulated in the high-expression group for functional and pathway analysis. The KEGG analysis showed that these upregulated genes were mainly involved in several key pathways, including the cell cycle, the AGE-RAGE signaling pathway, ECM-receptor interactions, complement and coagulation cascades, as well as p53 signaling. GO analysis indicated that these genes significantly influenced several biological processes, such as mitotic cell cycle phase transition, chromosome segregation, and nuclear chromosome segregation (**Figures 3A–C**). These findings suggest a strong association between many genes and the cell cycle. Key genes involved include PTTG1, CCNB1, CCNB2, CDKN2C, AURKB, CDK1, CCNA2, TGFB2, CDCA5, NDC80, CDC45, BUB1, WEE1, and MCM2 (**Figure 3C**). Additionally, we performed GSEA on the upregulated genes in the TUBA1B high-expression group, which again highlighted the cell cycle as a major pathway of interest (**Figure 3D**).

### 3.4 TUBA1B mediates malignant progression of glioblastoma by regulating the cell cycle

**Figure 4:**
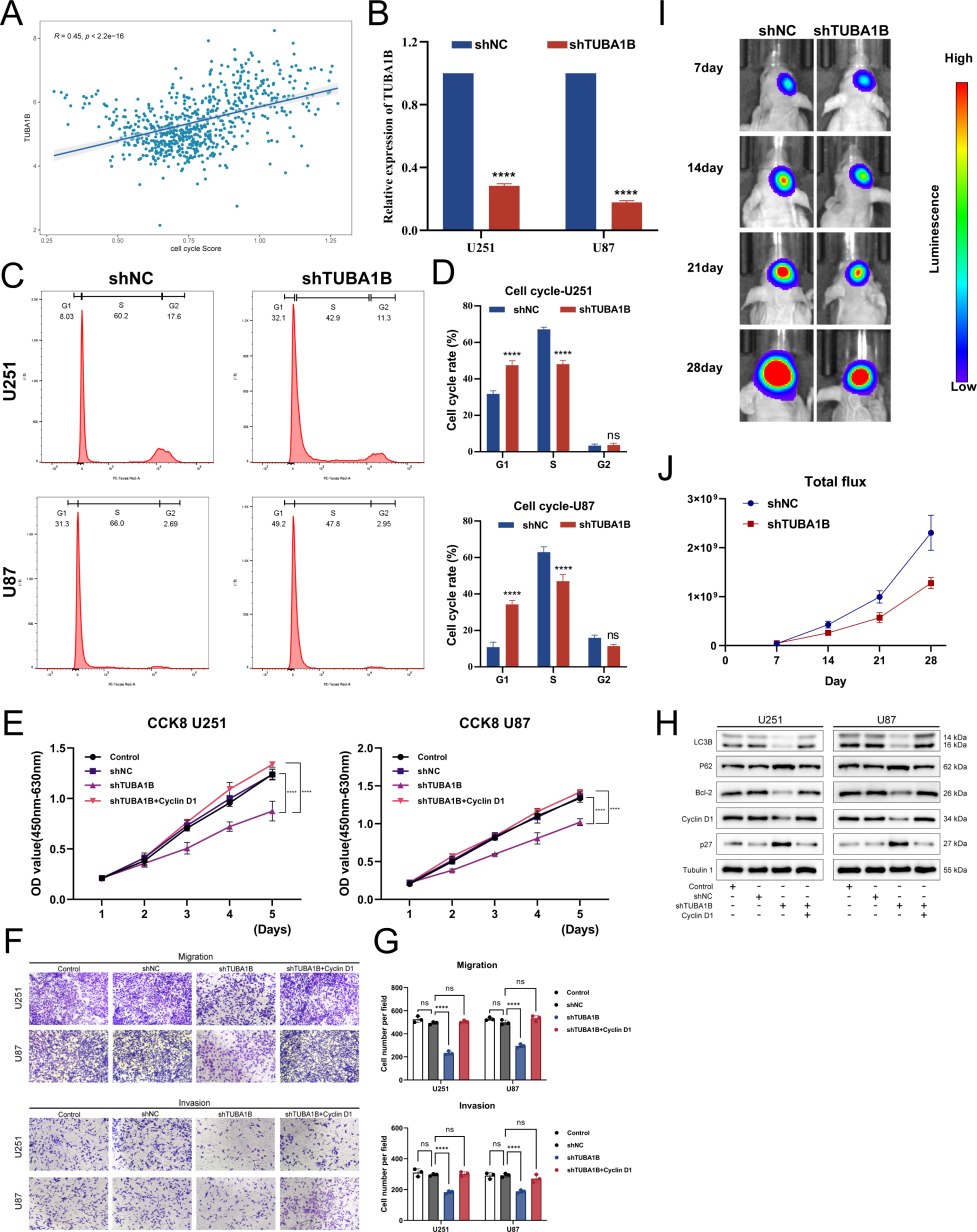
TUBA1B influences tumor malignancy progression. **(A)** Correlation analysis between TUBA1B expression and cell cycle score in glioma samples from the TCGA database. **(B)** qPCR analysis confirming the successful knockdown of TUBA1B in U251 and U87 cells. **(C, D)** Flow cytometry analysis of the cell cycle in U251 cells with TUBA1B knockdown. **(E)** CCK-8 assay showing the effect of TUBA1B knockdown on cell proliferation in U251 and U87 cells. **(F, G)** Migration and invasion assay in U251 and U87 cells with TUBA1B knockdown. **(H)** Western blot analysis showing the expression of Cyclin D1, p27, LC3B, and Bcl-2 in U251 and U87 cells with TUBA1B knockdown. **(I)** *In vivo* tumor growth analysis using a mouse xenograft model. **(J)** Statistical analysis of the total fluorescence intensity from the *in vivo* imaging.

The pathway analysis results suggest that TUBA1B may be associated with the cell cycle. Therefore, we performed a correlation analysis and found that the expression of TUBA1B was significantly positively correlated with the cell cycle score (**Figure 4A**). This finding prompted us to conduct a series of *in vitro* experiments to explore the role of TUBA1B in regulating cell cycle progression and its effect on glioma cell proliferation. First, we successfully knocked down TUBA1B expression in U251 and U87 cells (**Figure 4B**). Next, flow cytometry was used to analyze the cell cycle, and the results showed that knockdown of TUBA1B led to significant changes in the cell cycle distribution, particularly in the proportion of cells in the G1 and S phases. The percentage of cells in the G1 phase was significantly increased, while the proportion of cells in the S phase was significantly decreased (**Figures 4C, D**). These results suggest that TUBA1B may regulate cell proliferation by affecting the progression of the cell cycle. Furthermore, we evaluated cell proliferation using the CCK-8 assay. Knockdown of TUBA1B significantly reduced the proliferation rate of U251 and U87 cells (**Figure 4E**). Additionally, migration and invasion assays showed that TUBA1B knockdown significantly inhibited the migration and invasion capabilities of U251 and U87 cells (**Figures 4F, G**). However, the addition of the cell cycle activator Cyclin D1 partially restored the inhibitory effect of TUBA1B knockdown on cell migration and invasion, further validating that TUBA1B regulates glioma cell behavior through the cell cycle. Next, we performed Western blot analysis, which revealed that knockdown of TUBA1B led to a significant decrease in Cyclin D1 levels, while p27 protein levels were significantly increased (**Figure 4H**). This result further confirms the regulatory role of TUBA1B on key cell cycle proteins. Additionally, autophagy-related proteins such as LC3B and Bcl-2 also showed changes in expression, indicating that TUBA1B may also be involved in autophagy regulation. These effects were also partially reversed by Cyclin D1, supporting the involvement of the cell cycle in the regulation of TUBA1B’s role in glioma. Finally, we further validated the impact of TUBA1B on tumor progression using a mouse xenograft model. Fluorescence imaging results showed that tumor growth was significantly inhibited in the TUBA1B knockdown group (**Figure 4I**). Statistical analysis (**Figure 4J**) indicated that the total fluorescence intensity of the tumor in the TUBA1B knockdown group was significantly lower than that in the control group, further proving the oncogenic role of TUBA1B in glioma. In conclusion, TUBA1B regulates cell cycle progression and associated pathways, significantly affecting glioma cell proliferation, migration, and invasion. It also promotes tumor growth in the mouse xenograft model, suggesting that TUBA1B plays a crucial role in the progression of glioma.

### 3.5 TUBA1B affects the immune microenvironment of glioblastoma

**Figure 5:**
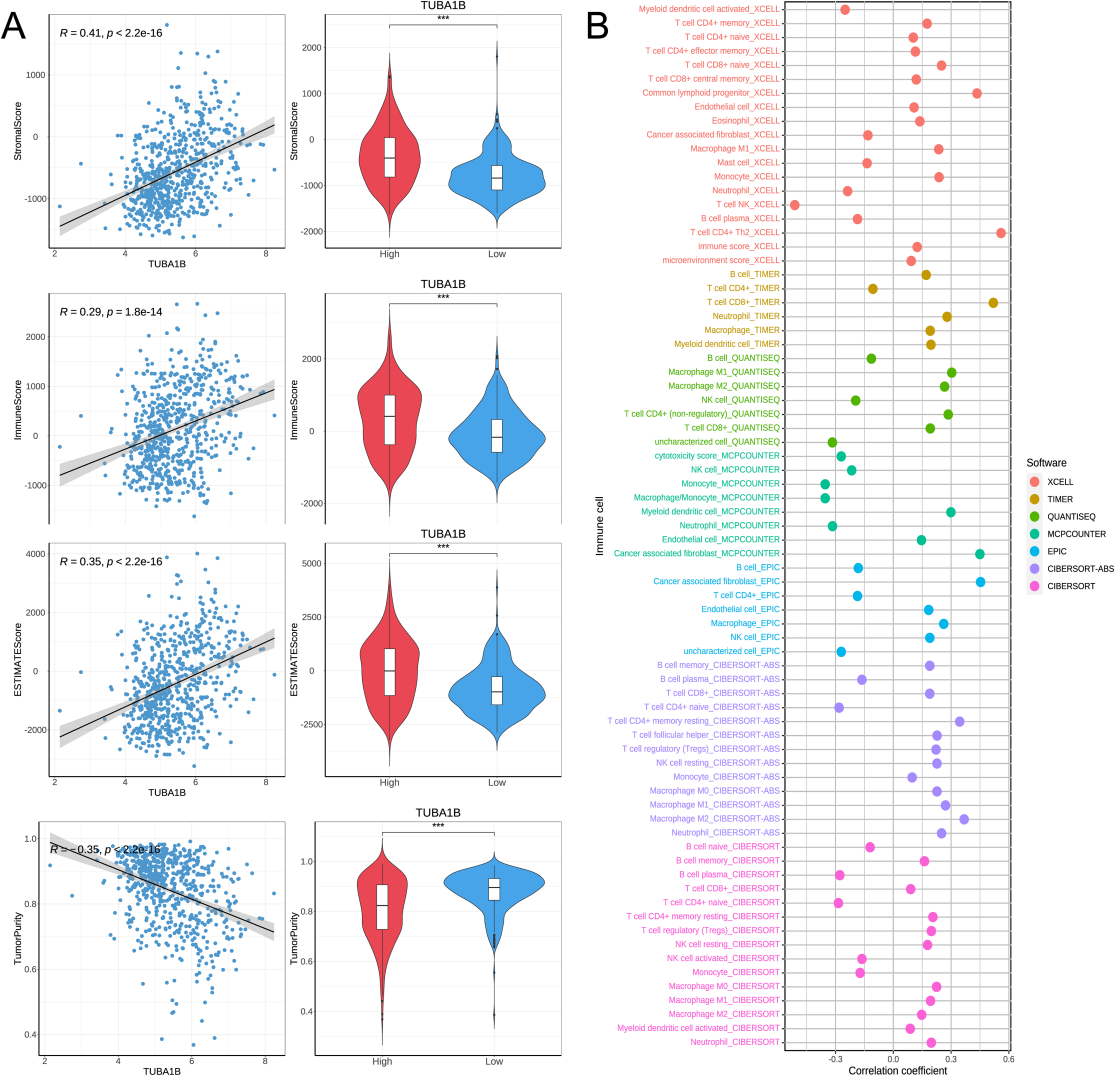
TUBA1B affects immune microenvironment in glioblastoma. **(A)** Comparison of Immune score, Estimate score, Stromal score, and Tumor purity between high and low TUBA1B expression groups. **(B)** Heatmap depicting significant differential immune responses between high and low TUBA1B expression groups using TIMER, CIBERSORT, CIBERSORT-ABS, QUANTISEQ, MCPCOUNTER, XCELL, and EPIC algorithms.

Based on correlation analyses and immune infiltration assessments, we investigated how TUBA1B affects the immune microenvironment in glioblastoma. First, we evaluated the correlation between TUBA1B expression and EstimateScore, ImmuneScore, StromalScore, and TumorPurity. In the high-expression TUBA1B group, the EstimateScore, ImmuneScore, and StromalScore were significantly elevated and positively correlated with TUBA1B expression (**Figure 5A**). The higher the expression of TUBA1B, the lower the TumorPurity, while there was a negative correlation between TUBA1B expression and TumorPurity (**Figure 5A**). Next, we used multiple algorithms (XCELL, QUANTISEQ, MCPCOUNTER, TIMER, CIBERSORT-ABS, EPIC, and CIBERSORT) to assess differences in immune infiltration between the high and low TUBA1B expression groups (**Figure 5B**). Our analysis focused on results with correlation coefficients greater than 0.3 to determine whether TUBA1B expression correlated with specific immune cell subtypes (**Supplementary Figure 3**). According to EPIC and MCPCOUNTER algorithms, TUBA1B expression and CAF infiltration are significantly correlated (R = 0.45, P < 2.2e-16). In the XCELL algorithm, the immune infiltration of T helper type 2 cells (CD4+Th2) also exhibited a strong positive correlation with TUBA1B expression (R = 0.56, P < 2.2e-16). The TIMER algorithm demonstrated that CD8^+^T cell infiltration was significantly positively correlated with TUBA1B expression (R = 0.52, P < 2.2e-16). Additionally, the QUANTISEQ algorithm revealed a positive correlation between M1-type macrophage infiltration and TUBA1B expression (R = 0.3, P = 1.8e-15), while the CIBERSORT-ABS algorithm showed a positive correlation between M2-type macrophage infiltration and TUBA1B expression (R = 0.37, P < 2.2e-16). By contrast, the MCPCOUNTER algorithm found that monocyte infiltration was significantly correlated with TUBA1B expression (R = -0.35, P < 2.2e-16), and in the XCELL algorithm, NK cell (natural killer cell) infiltration was also negatively correlated with TUBA1B expression (R = -0.51, P < 2.2e-16). The results indicate that TUBA1B potentially modulates tumor biology in human gliomas by affecting the immune microenvironment, especially through its impact on fibroblast infiltration and diverse immune cell types.

### 3.6 TUBA1B influences stemness and therapy response in glioma

**Figure 6:**
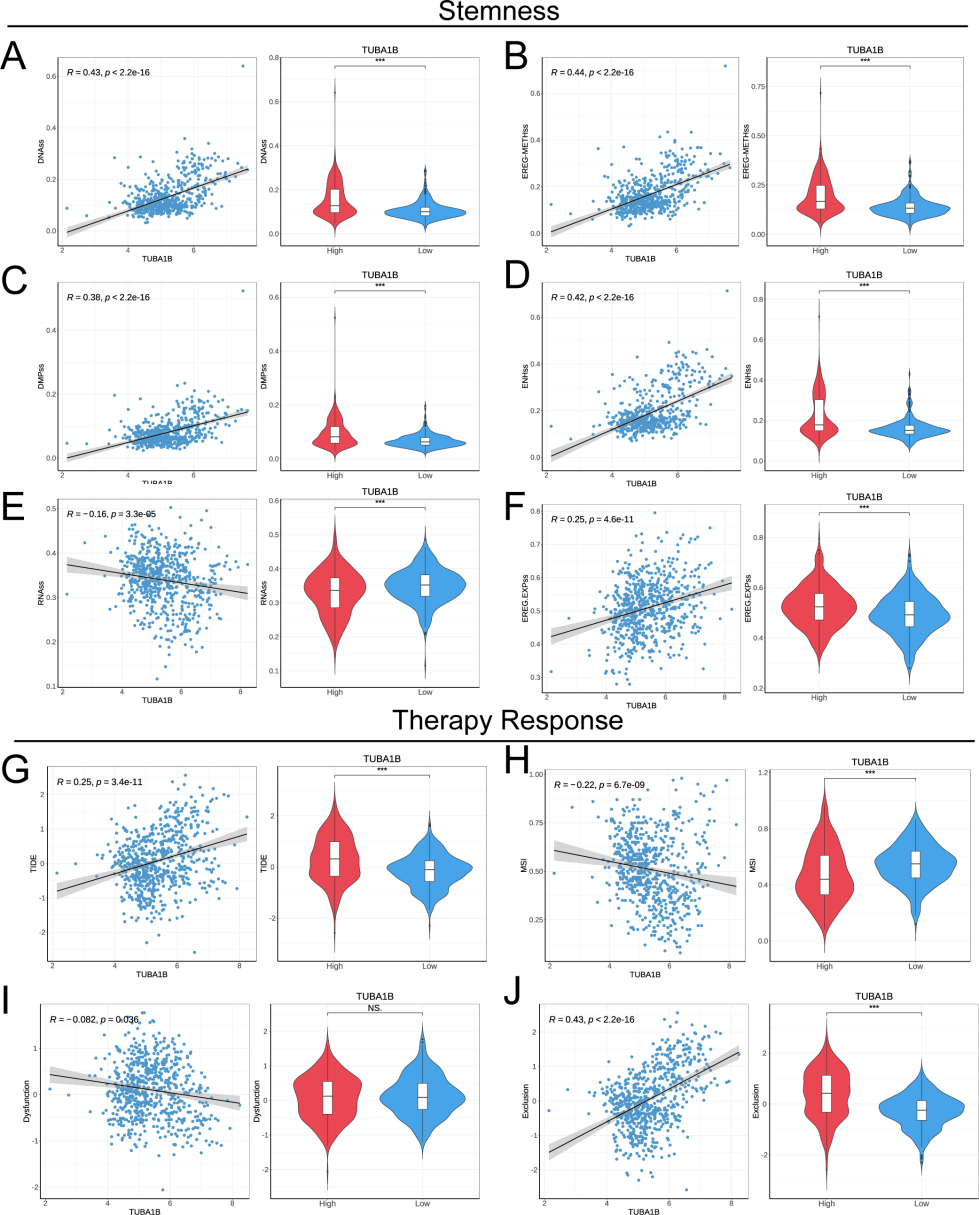
TUBA1B influences stemness and treatment response in glioma. **(A−F)** Correlation of tumor stemness with TUBA1B levels analyzed using DNAss, EREG-METHss, DMPss, ENHss, RNAss, and EREG.EXPss. **(G)** TIDE prediction scores between high and low TUBA1B expression groups in the TCGA dataset. **(H−J)** Comparison of responses to immunotherapy between high and low TUBA1B expression groups in the TCGA dataset.

To explore the impact of TUBA1B expression on tumor stemness in glioma, we conducted a Spearman correlation analysis. The results demonstrated that TUBA1B expression was significantly positively correlated with four tumor stemness indices (DNAss, EREG-METHss, DMPss, and ENHss), while it was significantly negatively correlated with RNAss and EREG.EXPss, all showing statistical significance (**Figures 6A–F**). Next, we used the TIDE (Tumor Immune Dysfunction and Exclusion) algorithm to assess the predictive ability of TUBA1B expression for immunotherapy benefits. High-expression TUBA1B had higher TIDE and Exclusion scores than low-expression TUBA1B, suggesting a higher immune escape potential. A lower MSI (Microsatellite Instability) score was also observed in the high-expression group, whereas no significant change was seen in the Dysfunction score. The results of a correlation analysis confirmed these findings, showing a significant positive correlation between TUBA1B expression, TIDE, and Exclusion, as well as a significant negative correlation with MSI (**Figures 6G–J**). These results suggest that high TUBA1B expression may promote tumor stemness and decrease sensitivity to immunotherapy in gliomas, highlighting the potential importance of TUBA1B in glioma progression and treatment response.

### 3.7 Intercellular crosstalk disrupts glioblastoma progression

**Figure 7:**
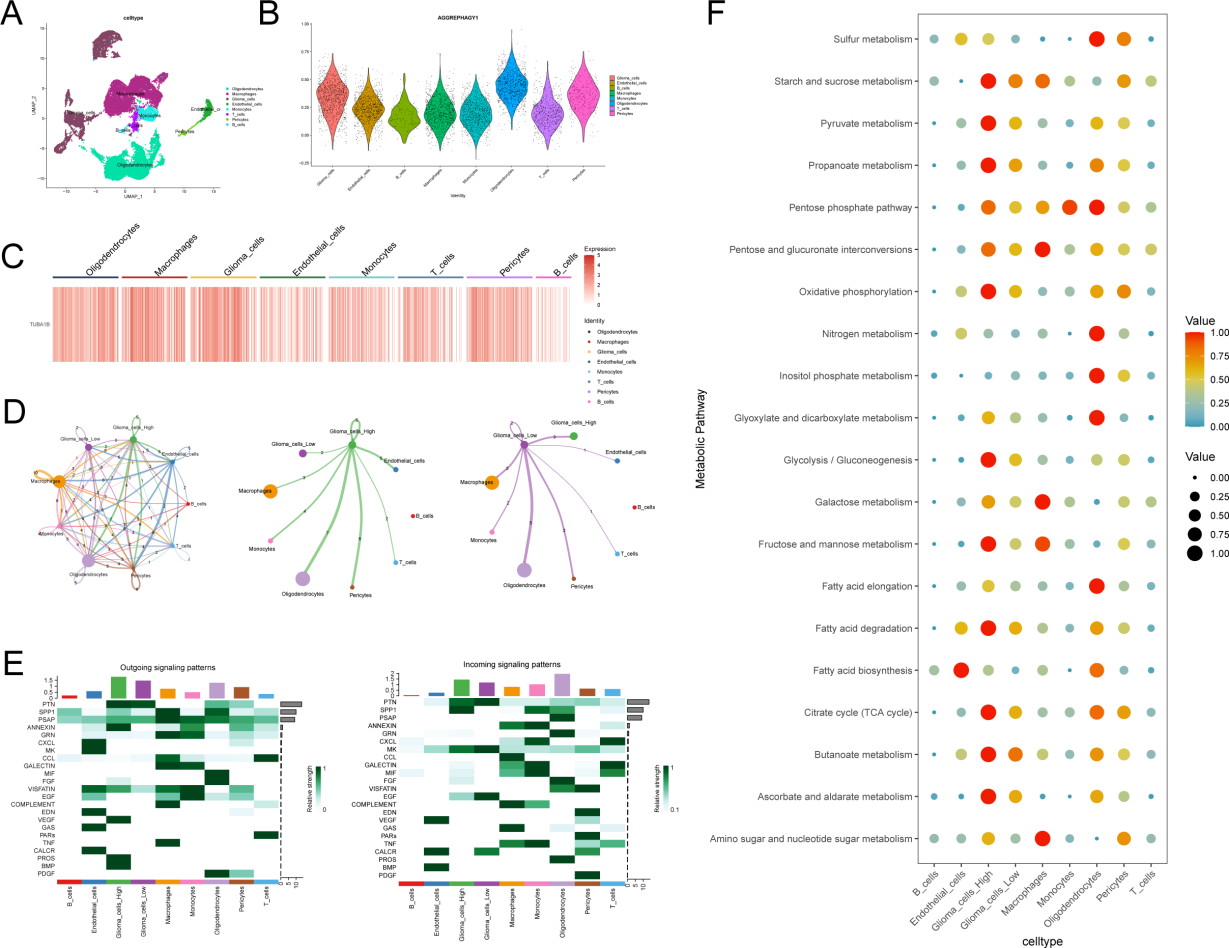
Analysis of TUBA1B in regulating glioma cell communication and metabolism within the tumor microenvironment. **(A)** UMAP plot used for cell type annotation in glioma and control samples. **(B)** Autophagy scores in different subsets of cells. **(C)** Heatmap of TUBA1B expression across different cell clusters. **(D)** Communication networks among different cell clusters and between high and low TUBA1B expression groups in gliomas. **(E)** Input and output signaling patterns of glioma cell communication in high and low TUBA1B expression groups. **(F)** Dot plot displaying activity of metabolic signaling pathways in different cell populations.

From six samples, we analyzed single cell sequencing data to understand TUBA1B’s role in the tumor microenvironment. After the initial screening, 23,520 cells were collected. Using the UMAP method for dimensionality reduction and unsupervised clustering, and with the help of known markers, the cells were classified into eight groups: Oligodendrocytes, Macrophages, Glioma cells, Endothelial cells, Monocytes, T cells, Pericytes, and B cells (**Figure 7A**; **Supplementary Figure 4**). We then calculated autophagy scores based on gene expression levels across these cell groups. As shown in **Figure 7B**, the results indicated that Oligodendrocytes and Glioma cells exhibited relatively high autophagy scores, suggesting that these cell groups may have high autophagic activity within the tumor microenvironment. Analysis of TUBA1B expression across different cell groups revealed that TUBA1B was predominantly expressed in Oligodendrocytes, Macrophages, Glioma cells, and Pericytes (**Figure 7C**). Based on this, we conducted a cell-cell communication analysis to clarify the interactions between TUBA1B-high-expressing cell groups. As expected, TUBA1B-high-expressing Oligodendrocytes, Macrophages, and Glioma cells displayed strong communication abilities (**Figure 7D**). Specifically, in Glioma cells, regardless of TUBA1B expression levels, these cells mainly interacted with Macrophages, Glioma cells, and Pericytes (**Figure 7D**). We then analyzed the main input and output signaling pathways of Glioma cells. For TUBA1B-high-expressing Glioma cells, signal output was primarily through the PTN, ANNEXIN, VEGF, PROS, and BMP pathways, while signal input occurred via the PTN, SPP1, and MK pathways. In contrast, TUBA1B-low-expressing Glioma cells mainly transmitted signals through the PTN pathway and received signals via the PTN, MK, EGF, and CALCR pathways (**Figure 7E**). Finally, we explored the metabolic pathways associated with Glioma cells. Both TUBA1B-high and TUBA1B-low Glioma cell groups were found to be involved in pathways related to starch and sucrose metabolism, propionate metabolism, oxidative phosphorylation, fatty acid degradation, and butyrate metabolism (**Figure 7F**). These findings suggest that TUBA1B may regulate glioma cell behavior by affecting intercellular communication and metabolic pathways within the tumor microenvironment.

## 4 Discussion

In glioma research, autophagy is regarded as a “double-edged sword.” On one hand, autophagy exacerbates tumor malignancy by promoting the maintenance and differentiation of glioma stem cells. This function can be attributed to the role of autophagy in protein degradation and cellular energy homeostasis (25). On the other hand, tumor development could be inhibited by autophagy via degrading waste, such as aggregated proteins (26). This dual role makes the regulation of autophagy a critical target for glioma therapy (27, 28). The cross-regulation between autophagy and the ubiquitin-proteasome system also plays a significant role in the growth and chemoresistance of glioma cells (19, 25). Aggrephagy, a selective form of autophagy that targets aggregated proteins for degradation, is pivotal in tumorigenesis and progression (29). Recent studies, particularly by Zhang et al. (30), have identified five aggrephagy-related genes (ARPS) and constructed prognostic signatures, validating their clinical relevance. These findings underline the importance of aggrephagy in gliomas, especially regarding the tumor microenvironment and prognosis.

In this comprehensive study, we have, for the first time, revealed the molecular subtypes of aggrephagy in gliomas and their complex interactions with the immune microenvironment and tumor progression. Through an integrated analysis of the TCGA database, two aggrephagy subtypes were identified: “immune-cold” and “immune-hot.” The latter is associated with poor survival outcomes, suggesting that aggrephagy may modulate tumor progression by influencing the immune microenvironment. Extensive immune cell infiltration in the “immune-hot” subtype may correspond with a pro-inflammatory state of the tumor, aligning with the intricate immune landscape of gliomas and indicating the potential influence of aggrephagy on immune evasion. However, it is important to note that while the “immune-hot” subtype shows significant immune infiltration, it may also be indicative of immune evasion mechanisms, and future studies should explore the precise immune modulatory role of aggrephagy and its interaction with immune checkpoints.

TUBA1B, a member of the tubulin family, is involved in cytoskeletal formation and cell division (31). In this study, TUBA1B was identified as a core gene of aggrephagy, showing potential as an independent prognostic marker. Machine learning analysis indicates that TUBA1B is a key driver of glioma progression, with high expression correlating with poor prognosis and aggressive tumor behavior. Moreover, TUBA1B has been implicated in poor prognosis and chemoresistance in various cancer types (32, 33). Our findings support these observations and suggest that TUBA1B’s role in glioma may be multifaceted, affecting not only tumor growth but also the tumor microenvironment, potentially enhancing immune evasion. Transcriptomic and functional analyses revealed a close association between TUBA1B overexpression and cell cycle regulatory genes such as Cyclin D1. Additionally, TUBA1B overexpression modulates various signaling pathways, including AGE-RAGE, ECM-receptor interactions, and complement and coagulation cascades. These pathways are associated with tumor growth, metastasis, and immune evasion in other cancer types (34–37), supporting the multifaceted role of TUBA1B in tumor biology. Studies have shown that a protein encoded by a short open reading frame in the TUBA1B gene plays a role in regulating tumor cell proliferation (38). Our *in vitro* experiments further validated the multifaceted role of TUBA1B in promoting cell proliferation and migration and inhibiting autophagy and apoptosis in gliomas, suggesting its potential as a therapeutic target. These findings underscore TUBA1B’s oncogenic potential and its critical role in glioma malignancy. However, future research should focus on developing specific inhibitors targeting TUBA1B, as well as understanding the broader molecular network through which it operates, including potential interactions with autophagy and immune pathways. Some studies have found that TUBA1B and its homolog TUBA1C are involved in regulating immune cell infiltration within the tumor microenvironment (39, 40). Our research reveals that high TUBA1B expression correlates significantly with decreased tumor purity and increased immune and stromal scores, possibly regulating tumor behavior by influencing immune infiltration. Notably, TUBA1B expression was found to correlate with a shift in immune cell composition, particularly in terms of macrophage infiltration, which may contribute to immune evasion mechanisms in gliomas. This highlights the need for future studies to investigate the interplay between TUBA1B and immune cell subsets in more detail. Finally, single-cell sequencing analysis provides new insights into the role of TUBA1B in cell-cell communication and metabolic pathways. TUBA1B is highly expressed in specific cell populations, such as oligodendrocytes and glioma cells, and is associated with extensive communication networks, indicating its potential collaborative regulatory role in gliomas.

Despite the compelling findings of our study, there are several limitations that must be addressed. First, the primary data used in this study, including TCGA, GEO, and CGGA datasets, are publicly available databases. Although these datasets are robust and widely used in glioma research, they are not exhaustive and may not fully represent the diversity of glioma patients in clinical settings. For example, the lack of detailed treatment regimens and patient follow-up data in some of these datasets may introduce bias in the survival analysis and clinical correlation. Additionally, our study primarily relied on bioinformatic analyses and computational tools to identify molecular signatures and relationships. While these methods are powerful, they cannot replace experimental validation, and we acknowledge that the predictive value of TUBA1B as a biomarker or therapeutic target must be further confirmed through *in vitro* and *in vivo* experiments. Furthermore, the retrospective nature of the data used in our analysis may limit the generalizability of our findings, and prospective studies are necessary to validate the clinical relevance of TUBA1B in glioma patients.

TUBA1B has shown promising potential as a prognostic biomarker for glioma patients, with higher expression levels correlating with poor survival outcomes. These findings suggest that TUBA1B could be an effective therapeutic target for glioma, providing a new avenue for glioma treatment. However, translating these findings into clinical practice will require further validation in preclinical and clinical settings. Combining TUBA1B inhibition with other therapeutic strategies, such as chemotherapy, radiation therapy, or immunotherapy, could also hold promise for improving treatment efficacy.

Furthermore, TUBA1B expression levels could be used to stratify glioma patients based on their risk of progression, enabling more personalized treatment approaches. Patients with high TUBA1B expression could benefit from more aggressive treatment regimens or experimental therapies targeting cell cycle regulators. Moreover, as TUBA1B is implicated in modulating the tumor microenvironment, future research could explore the synergy between TUBA1B inhibition and immunotherapy, which might enhance the immune response against glioma.

In summary, this study delineates the complex mechanisms of TUBA1B in gliomas, offering a new perspective on the role of aggrephagy in malignant tumors. Future in-depth experimental validation of the functions of TUBA1B, as well as its application in diverse clinical conditions, may propel the development of precision medicine and targeted therapies in gliomas.

## 5 Conclusion

This study deeply explores the biological characteristics and clinical significance of aggrephagy in gliomas. By analyzing the TCGA dataset, we identified two aggrephagy subtypes and revealed their differences within the tumor microenvironment through immune infiltration analysis. Among the highlighted genes, TUBA1B emerged as a key gene, demonstrating potential as an independent prognostic marker. *In vitro* functional experiments further confirmed that TUBA1B promotes the proliferation, migration, and invasion of glioma cells and is related to dynamic changes in the immune microenvironment. Single-cell sequencing analysis indicates that high TUBA1B expression is associated with specific intercellular communications and metabolic pathways, impacting tumor progression. Collectively, these findings underscore the vital role of TUBA1B in the occurrence, development, and treatment of gliomas, suggesting its potential as a clinical target. This provides a possible direction for precision medicine and new therapeutic strategies for gliomas.

## Data Availability

The data presented in this study are deposited in the following repositories: The TCGA data are available through the Genomic Data Commons (GDC) portal (https://portal.gdc.cancer.gov/), under the TCGA-GBMLGG cohort. The GEO dataset (GSE167960) can be accessed through the NCBI GEO database (https://www.ncbi.nlm.nih.gov/geo/). The CGGA data, including the mRNAseq_693, mRNAseq_325, and mRNA-array_301 datasets, are accessible through the CGGA database website (http://www.cgga.org.cn/).
