# Supplementary material for: The aggrephagy-related gene TUBA1B influences clinical outcomes in glioma patients by regulating the cell cycle
Source: Front Oncol. 2025 Feb 28;15:1531465. doi: 10.3389/fonc.2025.1531465 (PMC11906671; doi:10.3389/fonc.2025.1531465)
Supplement: Supplementary file 1 [file DataSheet1.docx]

**Supplementary Documents**

**
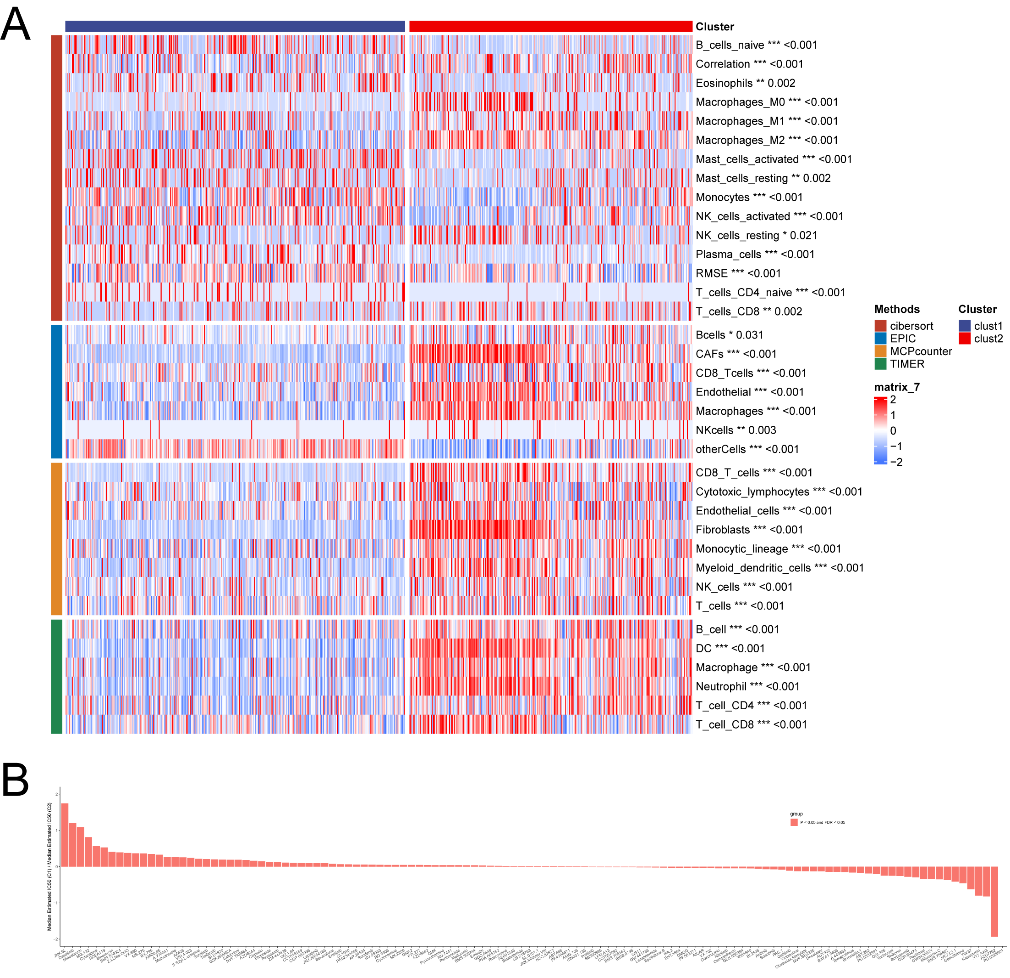
**

**Supplementary Figure 1. Immune infiltration scores between the two clusters calculated using different algorithms and different immune cells.**

**
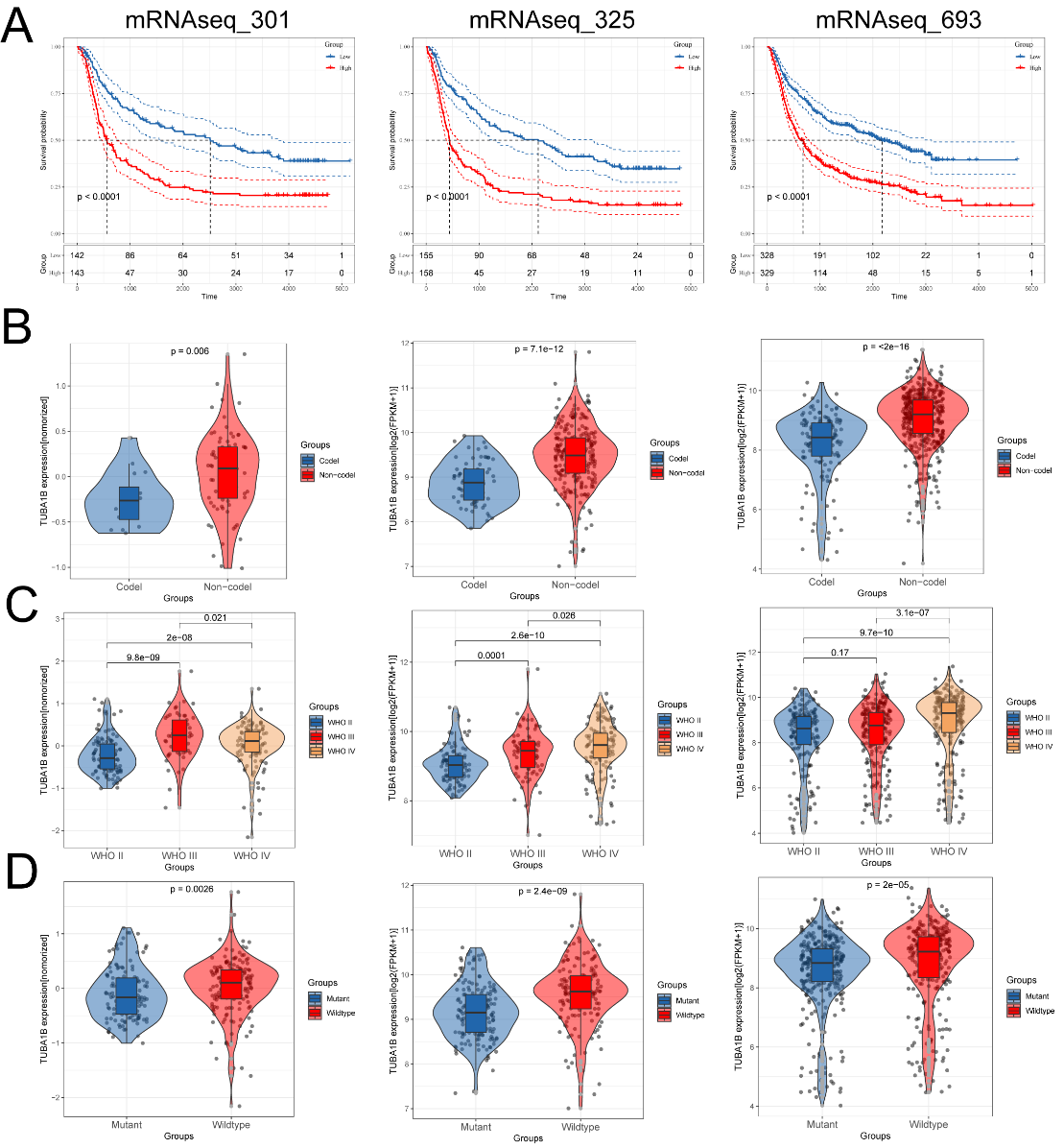
**

**
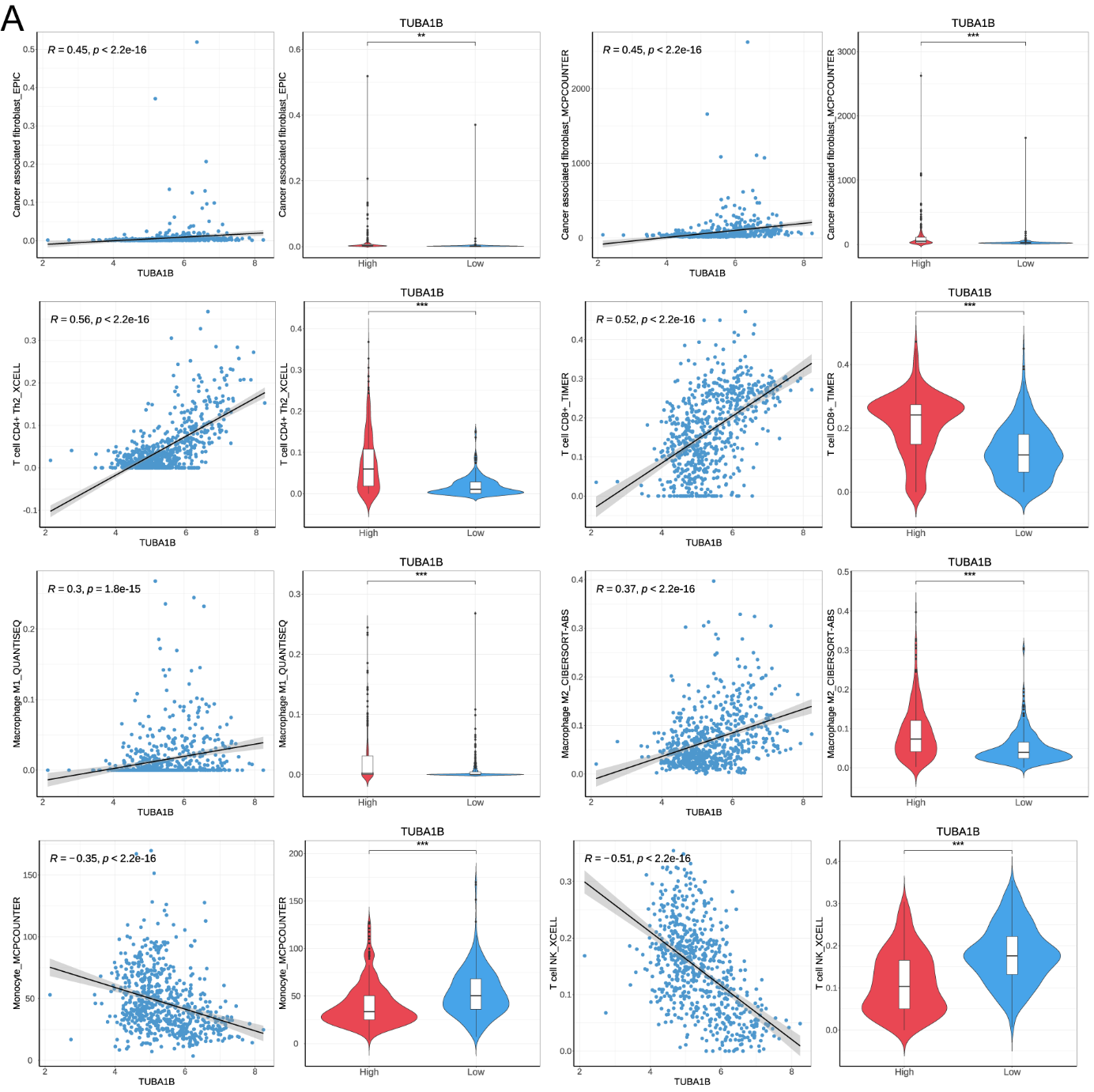
**

**Supplementary Figure 3. Correlation plots and violin plots of TUBA1B expression with immune cells showing correlation greater than 0.3.**

**
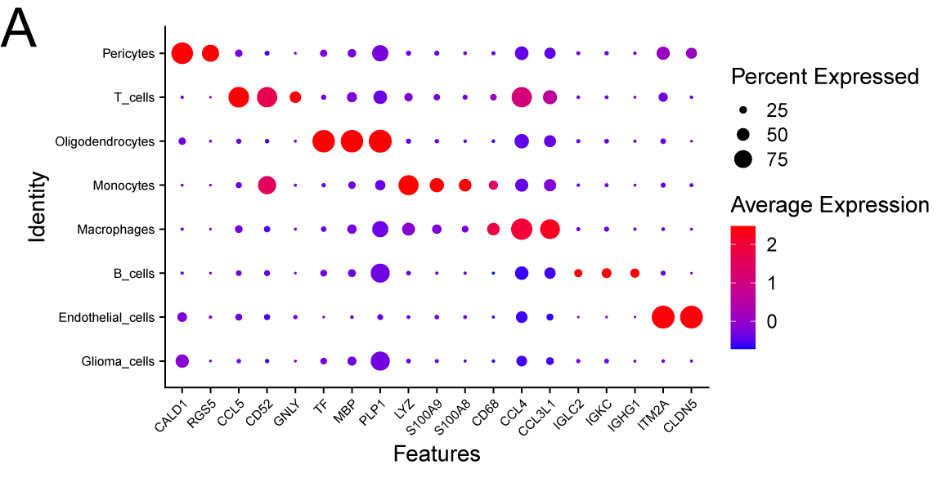
**

**Supplementary Figure 4. Bubble chart of gene marker differences in different cell clusters from single-cell sequencing.**

**WB original image supplement**

**
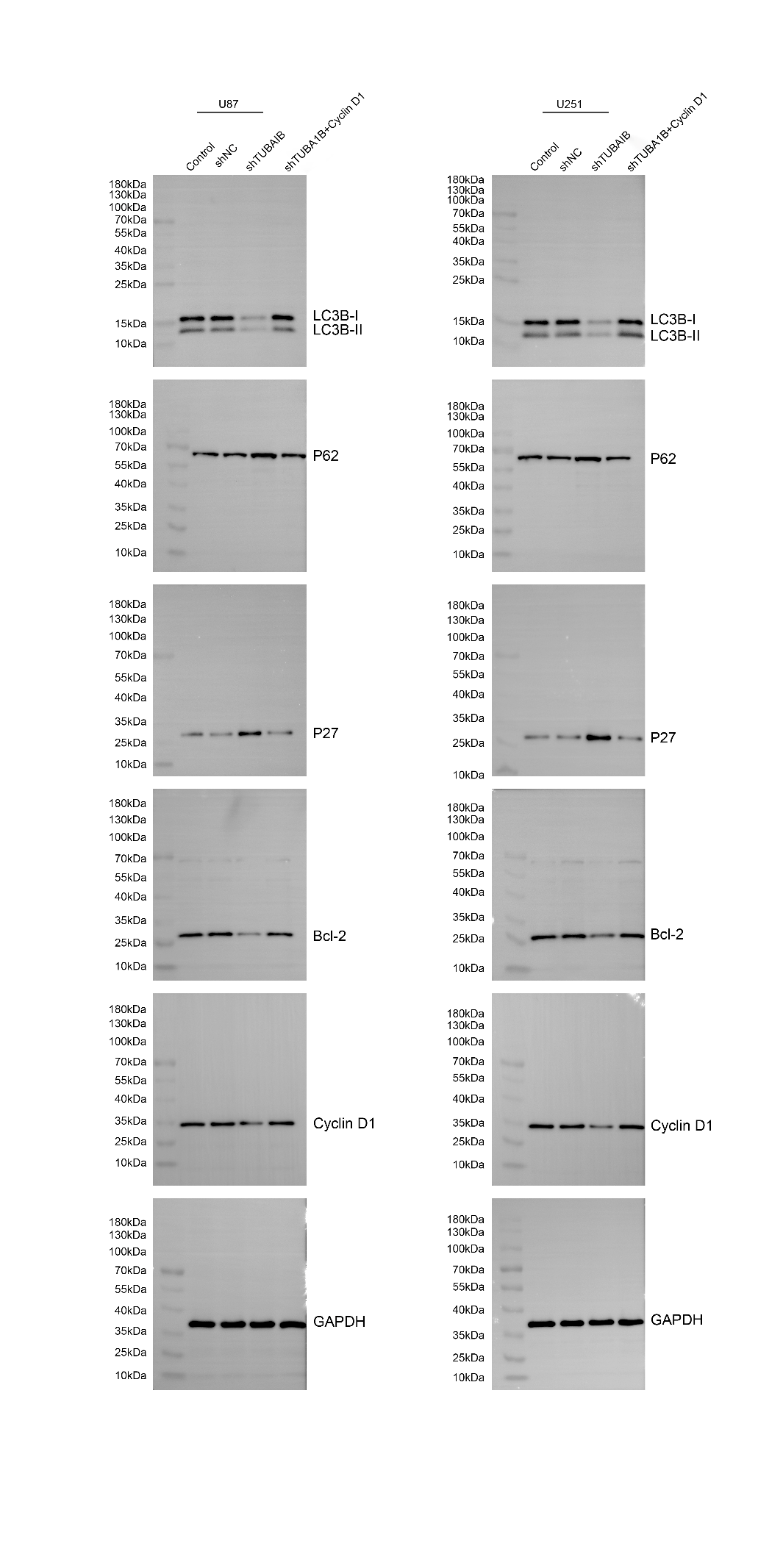
**
